# Effects of post-silking low temperature on the starch and protein metabolism, endogenous hormone contents, and quality of grains in waxy maize

**DOI:** 10.3389/fpls.2022.988172

**Published:** 2022-11-04

**Authors:** Jian Guo, Lingling Qu, Qi Wei, Dalei Lu

**Affiliations:** ^1^ Jiangsu Key Laboratory of Crop Genetics and Physiology/Jiangsu Key Laboratory of Crop Cultivation and Physiology/Agricultural College, Yangzhou University, Yangzhou, China; ^2^ Jiangsu Co-Innovation Center for Modern Production Technology of Grain Crops, Yangzhou University, Yangzhou, China; ^3^ Joint International Research Laboratory of Agriculture and Agri-Product Safety of the Ministry of Education, Yangzhou University, Yangzhou, China

**Keywords:** waxy maize, low temperature, grain physicochemical property, hormone content, starch and protein metabolism

## Abstract

Waxy maize has many excellent characteristics in food and nonfood industries. However, post-silking low temperature (LT) has severe limitations on its grain yield and quality. In this study, field and pot trials were conducted to investigate the effects of post-silking LT on the physiological, biochemical, and functional characteristics of two waxy maize grains. The field and pot trials were performed with sowing date and artificial climate chamber, respectively, for LT treatment from silking stage to maturity. Results in pot trial were used to explain and validate the findings in field trial. Compared with the ambient treatment, the LT treatment significantly reduced kernel weight during the grain filling stage (*P* < 0.05). LT treatment in both environments resulted in an average decrease in dry weight of SYN5 and YN7 at maturity by 36.6% and 42.8%, respectively. Enzymatic activities related to starch and protein biosynthesis decreased under the LT treatment during the filling stage, accompanied by a decrease in the accumulation amounts and contents of soluble sugar and starch, and a decrease in protein accumulation amount. Meanwhile, the contents of abscisic acid, indole-3-acetic acid, and gibberellin 3 in grains decreased under the LT treatment during the filling stage. Peak, trough, breakdown, final, and setback viscosities of grains decreased by LT. LT treatment decreased the gelatinization enthalpy of grains and increased the retrogradation percentage. In conclusion, post-silking LT stress altered the content of grain components by inhibiting the production of phytohormones and down-regulating the enzymatic activities involved in starch and protein metabolism, which resulted in the deterioration of grain pasting and thermal properties.

## Introduction

Low temperature (LT) is an important abiotic stress that seriously affects the production and development of global cereal crops (Huang et al., 2021; Zhang et al., 2021). Several studies have found that the post-silking/grain filling stage is very sensitive to LT during the growth cycle of cereal crops (Thakur et al., 2010; Luo, 2011). LT stress at this stage could slow down plant growth and reduce the grain filling rate, grain weight and grain setting rate, ultimately decrease the grain yield and quality (Kabir et al., 2014; Koga et al., 2015; Ali et al., 2021).

Starch and protein are important components in grains, and the dynamics of their content and accumulation determines the yield of cereal crops (Lu et al., 2014a; Huang et al., 2021; Zhang et al., 2021). In cereal grains, starch and protein biosynthesis is a complex process determined by the coordinated action of enzymes (Marschner, 2011; Ran et al., 2020). LT at the booting stage reduces the enzymatic activities of ADP-glucose pyrophosphorylase (ADPase), soluble starch synthase (SSS), and starch branching enzyme (SBE) and decreases the content and accumulation of starch, which consequently decline wheat grain dry matter accumulation (Zhang et al., 2021). A previous study found that LT during the flowering period alters the protein content and grain quality by affecting the source-sink relationship in rice (Huang et al., 2021). Meanwhile, phytohormones play essential roles in promoting and regulating cold tolerance of plants (Khan et al., 2017). The mutual regulation of endogenous hormones and enzymes and the balance of endogenous hormones play a crucial role in the grain filling process and the dry matter accumulation in grains (Yu et al., 2020). A previous study confirmed that LT at the booting stage induces a decrease in auxin and gibberellins in young ears of winter wheat, which alters the activities of enzymes involved in sucrose metabolism and cause yield loss (Zhang et al., 2019). Changes in endogenous hormones under LT stress are involved in regulating not only the grains development but also the plants LT resistance (Eremina et al., 2016; Gao J. et al., 2018). Furthermore, changes in the contents of grain components (i.e., sugar, starch, lipid, and protein) of cereal crops can alter the starch crystallinity and starch granule morphological characteristics, thereby affecting the thermal and pasting properties of flour (Lu and Lu, 2013; Lu et al., 2014b; Lin et al., 2020). Liu et al. (2019) found that LT during the jointing and booting stages increase protein content and decrease starch content in wheat grains, resulting in poor appearance quality. A study has reported that LT during the reproductive period causes deterioration of grain appearance, milling, and cooking quality traits in rice (Siddik et al., 2019). LT stress during the grain filling stage deteriorates flour functionality and eating quality in rice by increasing the amylose content and affecting the crystalline structure and pasting property of starch (Hu et al., 2020).

Waxy maize (*Zea mays* L. var. *ceratain* Kulesh), also known as sticky maize, features high viscosity and digestibility. Waxy maize has high economic, nutritional, and processing value; thus, its production has increased dramatically in recent decades (Singh et al., 2006; Lei et al., 2016; Yang L. et al., 2021). It is also an important industrial raw material in the textile, adhesive, brewing, and paper industries (Klimek-Kopyra et al., 2012; Liu et al., 2021). In China, summer maize is the second largest among six maize ecological regions and is seeded in late May and June after harvesting wheat and canola (Shu et al., 2021). The widely planted summer waxy maize frequently experiences high temperatures during the grain formation stage of its life cycle, resulting in reduced grain yield and quality (Lu et al., 2014b). Thus, postponing the sowing date of summer maize has become an important cultivation measure to avoid extreme heat damage (Tao et al., 2016; Lv et al., 2020). However, postponing the sowing date of summer maize is inappropriate because it increases the risk of chilling injury in the later growth stage (Tsimba et al., 2013; Tian et al., 2019). For summer maize in the North China Plain sown from June to July, the grain filling rate decreases rapidly because of the decrease in temperature and solar radiation during the late growth stage (late September), which consequently affects yield (Gao Z. et al., 2018). At present, few studies have documented the effects of LT stress at the late growth stage on grain development and quality formation in waxy maize. Therefore, understanding the effects of LT stress on the physiological, biochemical, and functional properties of waxy maize grains is important to increase yield and quality.

In this study, two waxy maize varieties were used in pot and field experiments to conduct LT experiments during the post-silking stage of waxy maize. The main objective was to investigate the effects of the post-silking LT on starch and protein metabolism, endogenous hormone contents, and function properties in grains. The results of these two experiments can provide a basis for studing the effect of LT on the grain development and quality of waxy maize. Combined field and pot trials are important for developing adaptive strategies for maize production under future climate conditions.

## Materials and methods

### Experimental design

The experiments were carried out at the experimental farm of Yangzhou University (Yangzhou, China) from July to November 2020. Two waxy maize varieties, Suyunuo5 (SYN5) and Yunnuo7 (YN7), provided by Jiangsu Yanjiang Institute of Agricultural Sciences (Nantong, China) and widely planted in Southern China, were as materials.

The combination of pot and field planting methods was used in our experiment. For the pot trial, plants were placed in pots 38 cm in height and 43 cm in diameter. Potting soil was sandy loam soil obtained from the 0–20 cm tillage layer of the experimental farm and had an organic matter content of 9.87 g/kg, a total nitrogen content of 1.12 g/kg, and available nitrogen, phosphorus, and potassium contents of 83.78, 7.14, and 68.51 mg/kg, respectively. Three waxy maize seeds were sowed on July 1, and one plant was retained at the six-leaf stage. After planting, 10 g commercial fertilizer (N:P_2_O_5_:K_2_O=15%:15%:15%) was applied to each pot, with an additional 6.6 g of urea (N= 46%) applied at the six-leaf stage. The pots were placed in the experimental plots until the maize silking stage. After manual pollination, these pots were moved into a controlled climatic chamber on the same day for the following temperature treatments: ambient temperature (AT), 28°C (light)/20°C (dark); low temperature (LT), 23°C (light)/15°C (dark). The photoperiod was 12 h (light, 06.00–18.00 h)/12 h (dark, 18.00–06.00 h). There were 50 pots for each temperature treatment, and these pots were stored in the chambers until maturity.

**Figure 1:**
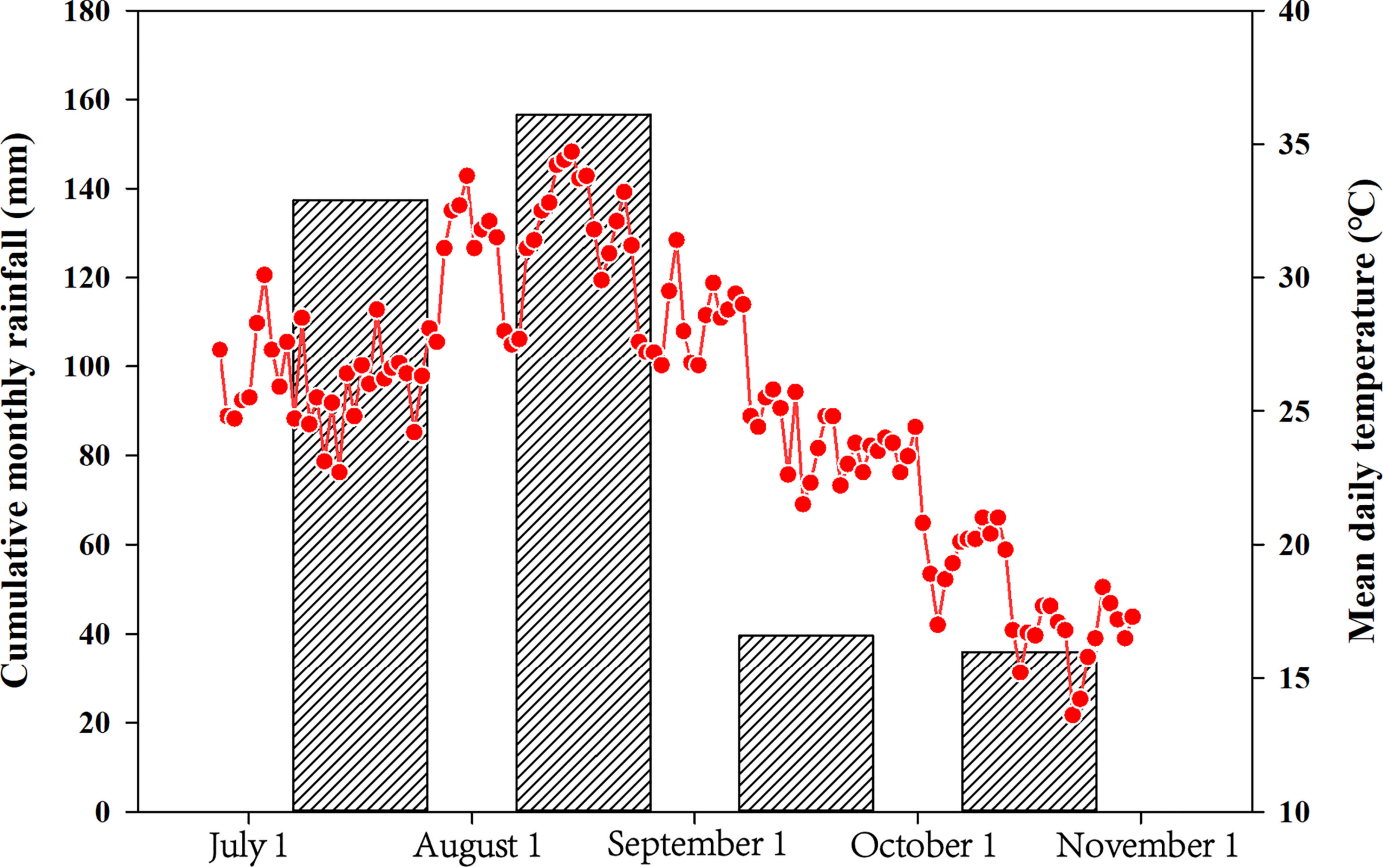
Average daily temperatures and cumulative monthly rainfall during the waxy maize-growing seasons from July1 to October 31.

For the field trial, the two waxy maize varieties were sown on July 1 (AT treatment) and August 1 (LT treatment) with a density of 60,000 plants/ha. Three replications with a random complete block design were prepared in the present experiment. The soil properties of the field trial were consistent with those of the pot trial. Each plot area was 24 m^2^ (3.2 m × 7.5 m) with a total of 12 plots. Meanwhile, 500 kg/ha commercial fertilizer (N:P_2_O_5_:K_2_O=15%:15%:15%) and 150 kg/ha urea (N=46%) were applied at the planting and jointing stages, respectively. The meteorological data of the field trial in the growing season in this study are summarized in **Figure 1**. The post-silking stage for the early and late sowing data was September 2 − October 8 (with a mean daily temperature 24.2°C) and September 29 – November 11 (with a mean daily temperature 17.7 °C), respectively. The post-silking cumulative monthly rainfall amounts were 39.6 and 36.0 mm in the early and late sowing dates, respectively.

### Sampling and grain yield determination

From 5 days after pollination (DAP), three uniform ears of each treatment were selected every 5 days until maturity. Approximately grains at the middle position were stripped from the ears, frozen in liquid nitrogen, and then stored at −80°C to analyze enzymatic activity and endogenous hormone content. Some remainder kernels were weighed to obtain the kernel fresh weight (mg). These kernels were fixed at 105°C for 30 min, dried to constant weight at 60°C, and then weighed to obtain the kernel dry weight (mg). All measurements were performed in triplicate.

### Soluble sugar, starch, and protein extraction

After drying, the grains at different stages (5, 10, 15, 20, 25, 30 DAP, and maturity) of each treatment were ground into a powder and passed through a 100-mesh (0.149 mm) sieve for physicochemical analysis. The contents of soluble sugar (mg/g) and starch (mg/g) in the grains were determined with an anthrone-sulfuric acid method (Hansen and Møller, 1975). The nitrogen content in the grains was determined using the Kjeldahl method (AACC International Approved Method 46-10.1), and protein content was calculated using the formula: protein content (mg/g) = nitrogen content × 6.25 (AACC International, 1990). Soluble sugar, starch, and protein accumulation (mg/kernel) were calculated using the formula: soluble sugar accumulation = kernel dry weight × soluble sugar content, starch accumulation = kernel dry weight × starch content, and protein accumulation = kernel dry weight × protein content, respectively. All measurements were performed in triplicate.

### Enzymatic activity assays for soluble sugar, starch, and protein biosynthesis

The activities of sucrose phosphate synthase (SPS), sucrose synthase (SuSy), AGPase, SSS, SBE, starch-debranching enzyme (SDBE), nitrate reductase (NR), glutamine synthetase (GS) and glutamate synthase (GOGAT) in grains at different stages (5, 10, 15, 20, 25 and 30 DAP) were determined by enzyme-linked immunosorbent assay (ELISA) using reagent kits ml10724, ml10561, ml20341, ml10983, ml076664, ml10726, ml076501, ml076529, and ml076499, respectively, which were obtained from Shanghai Enzyme-linked Biotechnology Co., Ltd (Shanghai, China) (Guo et al., 2021). All enzymatic activities were performed in triplicate.

### Quantification of endogenous hormone content

The contents of abscisic acid (ABA), indole-3-acetic acid (IAA), and gibberellin 3 (GA_3_) in grains at different stages (5, 10, 15, 20, 25, and 30 DAP) were determined by ELISA reagent kits ml077235, ml077231 and ml077232, respectively. These ELISA kits were purchased from Shanghai Enzyme-linked Biotechnology Co., Ltd (Shanghai, China) (Han et al., 2022). All endogenous hormones contents were performed in triplicate.

### Pasting property

The grains at mature stage were dried and ground with a high-speed disintegrator and passed through a 100-mesh sieve, and then the pasting property of the flour was determined. The pasting property of the waxy maize flour was measured by using a rapid viscosity analyzer (RVA-3D; Newport Scientific, Warriewood NSW, Australia) according to the method of Lu and Lu, 2013. The mature flour of 2.8 g (dry weight) was mixed with 25.2 mL of distilled water in an aluminum crucible. There are three steps involved in this process: firstly, the mixture sample was heated at 50°C for 1 min; secondly, the mixture sample was heated from 50 to 95°C at 12°C/min, maintained at 95°C for 2.5 min, and was cooled to 50°C at 12°C/min; thirdly, the mixture sample was cooled to 50°C at 12°C/min for 1 min. The paddle rotated at 960 rpm for 10 s and then decreased to 160 rpm. The pasting properties included peak viscosity (PV), trough viscosity (TV), breakdown viscosity (BD = PV - TV), final viscosity (FV), setback viscosity (SB = FV - PV) and pasting temperature (*P*_temp_). All of the measurements were performed in triplicate.

### Thermal property

The samples that were used for determining the pasting property were also used for the analysis of thermal property. The thermal characteristics of the waxy maize flour were measured by using a differential scanning calorimetry (DSC, Model 200 F3 Maia, NETZSCH, Germany) according to the method of Lu and Lu, 2013. The mature flour 5.0 mg (dry weight) was mixed with 10 μL of distilled water in an aluminum crucible and sealed hermetically. The mixture sample was equilibrated at 4°C for 24 h. Then the mixture sample was heated from 25°C to 100°C at 10 °C min^-1^. The gelatinization enthalpy (Δ*H*_gel_), onset temperature (*T*_o_), peak gelatinization temperature (*T*_p_), and conclusion temperature (*T*_c_) were obtained through data recording software. After thermal analysis, the samples were stored at 4°C for 7 d for the measurement of retrogradation enthalpy (Δ*H*_ret_) and retrogradation percentage (%*R* = 100 × Δ*H*_ret_/Δ*H*_gel_). All of the measurements were performed in triplicate.

### Statistical analysis

Statistical analyses of experimental data were performed using ANOVA in SPSS v. 19.0 (IBM, Armonk, NY, USA). The levels of statistical significance were determined using the least significant difference at the *P* < 0.05 level. Figures were made by GraphPad Prism 8 (GraphPad, San Diego, CA, USA). The correlation coefficient was calculated using Pearson’s correlation coefficient, and software R (v.3.6.1) was used to generate heatmaps of the data.

## Results

### Dynamic changes in kernel development under low temperature

**Figure 2:**
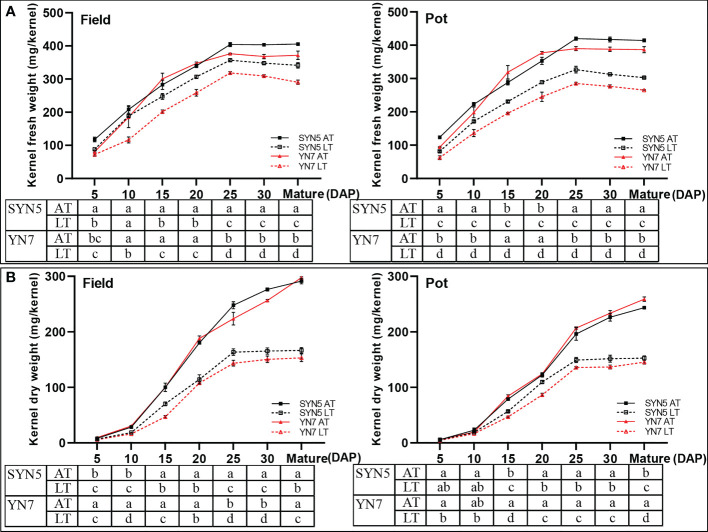
Effect of post-silking LT on the kernel fresh and dry weights of waxy maize during grain filling stage. **(A)**, kernel fresh weight; **(B)**, kernel dry weight. Error bars denote standard errors from three replicates, and different letters at the same sampling date indicate significant difference at *P* < 0.05.

Post-silking LT significantly reduced the fresh and dry weights of grain (*P* < 0.05) during kernel development compared with the AT treatment in both varieties and experimental environments. In the field trial, the kernel fresh weight during kernel development in the LT treatment of SYN5 and YN7 decreased by 9.4%–24.9% and 19.9%–34.1%, respectively, compared with that in the AT treatment (**Figure 2A**). In the pot trial, for SYN5 and YN7, the kernel fresh weights under the LT treatment were 10.5%–37.1% and 26.9%–38.6% lower than those under the AT treatment, respectively (**Figure 2A**). The kernel dry weights of SYN5 and YN7 from 5 DAP to maturity in the field trial decreased by 15.8%−40.1% and 35.9%−46.5%, respectively, compared with the AT treatment (**Figure 2B**). In the pot trial, the decreases in kernel dry weight of SYN5 and YN7 were 5.9%–37.3% and 14.4%–43.9% under the LT treatment, respectively.

### Dynamic changes in grain soluble sugar, starch, and protein accumulation under low temperature

**Figure 3:**
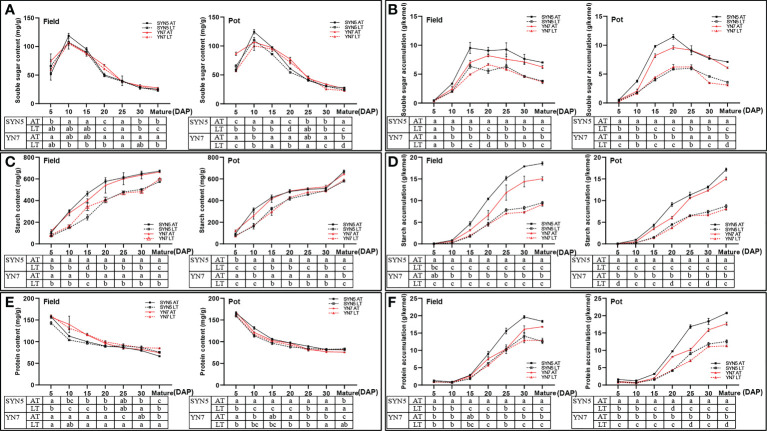
Effect of post-silking LT on the soluble sugar, starch and protein contents and accumulation in waxy maize grains during grain filling stage. **(A)**, soluble sugar content; **(B)**, soluble sugar accumulation; **(C)**, starch content; **(D)**, starch accumulation; **(E)**, protein content; **(F)**, protein accumulation. Error bars denote standard errors from three replicates, and different letters at the same sampling date indicate significant difference at *P* < 0.05.

The soluble sugar contents of both varieties and all samples initially increased and then decreased with kernel development (**Figure 3A**). Compared with the AT treatment, the LT treatment significantly decreased the soluble sugar content in the field trial at maturity in both varieties but did not affect the other stages (*P* < 0.05). In addition, the LT treatment significantly decreased the soluble sugar contents of SYN5 in the pot at 10, 15, 20 DAP, and maturity and those of YN7 at 5, 20, 30 DAP, and maturity. The amount of soluble sugar accumulation gradually increased with the advancement of grain filling and then decreased from 25 DAP to maturity (**Figure 3B**). Meanwhile, the LT treatment significantly decreased the soluble sugar accumulation in both varieties and trials (*P* < 0.05). At maturity, the reduction rates of the soluble sugar accumulation of SYN5 and YN7 under the LT treatment were 45.9% and 41.3% in the field trial, respectively, and 49.5% and 49.1% in the pot trial, respectively.

The starch content and accumulation of both varieties in all the treatment groups gradually increased with kernel development (**Figures 3C, D**). Compared with the AT treatment, the LT treatment during the grain filling stage significantly decreased starch content and accumulation in both varieties and environments (*P* < 0.05). At maturity, the starch contents of SYN5 and YN7 under the LT treatment decreased by 14.6% and 9.7% in the field trial, respectively. In the pot trial, the starch contents of SYN5 and YN7 at maturity were 13.4% and 8.5% lower under the LT treatment than under the AT treatment (**Figure 3C**). Similarly, the starch accumulation at maturity of SYN5 under the LT treatment decreased by 48.9% and 49.2% in the field and pot trials, respectively, and the starch accumulation at maturity of YN7 decreased by 39.5% and 46.5%, respectively (**Figure 3D**).

The protein content decreased during kernel development for all treatments in both varieties (**Figure 3E**). Compared with the AT treatment, the LT treatment significantly increased the protein content of SYN5 at 30 DAP and maturity and that of YN7 at 25 DAP and maturity in the field trial (*P* < 0.05). At maturity, the protein contents of SYN5 and YN7 under the LT treatment increased by 1.5% and 1.3%, respectively. In the pot trial, the protein contents of SYN5 and YN7 at maturity increased by 2.2% and 2.8%, respectively, under the LT treatment. By contrast, the LT treatment significantly reduced protein accumulation during grain filling in both varieties and environments (*P* < 0.05) (**Figure 3F**). In the end, the LT treatment reduced the protein accumulation of SYN5 and YN7 by 31.2% and 23.4% in the field trial at maturity, respectively, and by 39.6% and 36.3% in the pot trial, respectively.

### Effect of low temperature on the activities of enzymes involved in soluble sugar and starch syntheses

**Figure 4:**
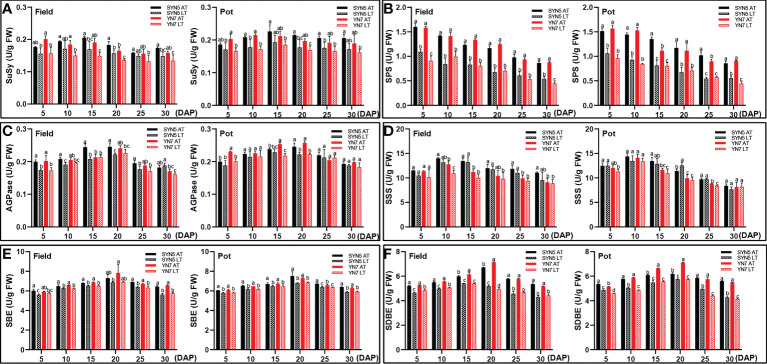
Effect of post-silking LT on the enzymatic activities of soluble sugar and starch synthase in grains during grain filling stage. **(A)**, SPS (sucrose phosphate synthase); **(B)**, SuSy (sucrose synthase); **(C)**, AGPase (ADP-glucose pyrophosphorylase); **(D)**, SSS (soluble starch synthase); **(E)**, SBE (starch branching enzyme); **(F)**, SDBE (starch-debranching enzyme). Error bars denote standard errors from three replicates, and different letters above the bars in same sampling date are significantly different at *P* < 0.05.

In the field trial, the LT treatment decreased the SuSy activity of SYN5 at 15, 20, and 30 DAP and that of YN7 during grain filling (**Figure 4A**). In the pot trial, the LT treatment decreased the SuSy activity of SYN5 at 10, 20, and 25 DAP and that of YN7 at 5, 10, and 20 DAP (**Figure 4A**). Compared with those under the AT treatment, the activities of SPS, SBE, and SDBE under the LT treatment significantly reduced during kernel development in both varieties and experimental environments (*P* < 0.05; **Figures 4B, E, F**). The AGPase of SYN5 in the field trial significantly decreased under the LT treatment during kernel development, and that of YN7 decreased at 5 and 20 DAP (*P* < 0.05; **Figure 4C**). The LT treatment in the pot trial almost unaffected AGPase in both varieties. Meanwhile, the SSS activities of both varieties were almost unaffected by the LT treatment (**Figure 4D**). On average, the enzymatic activities related to the soluble sugar and starch syntheses of SYN5 decreased less than YN7 under LT treatment.

### Effect of low temperature on the activities of enzymes involved in protein synthesis

**Figure 5:**
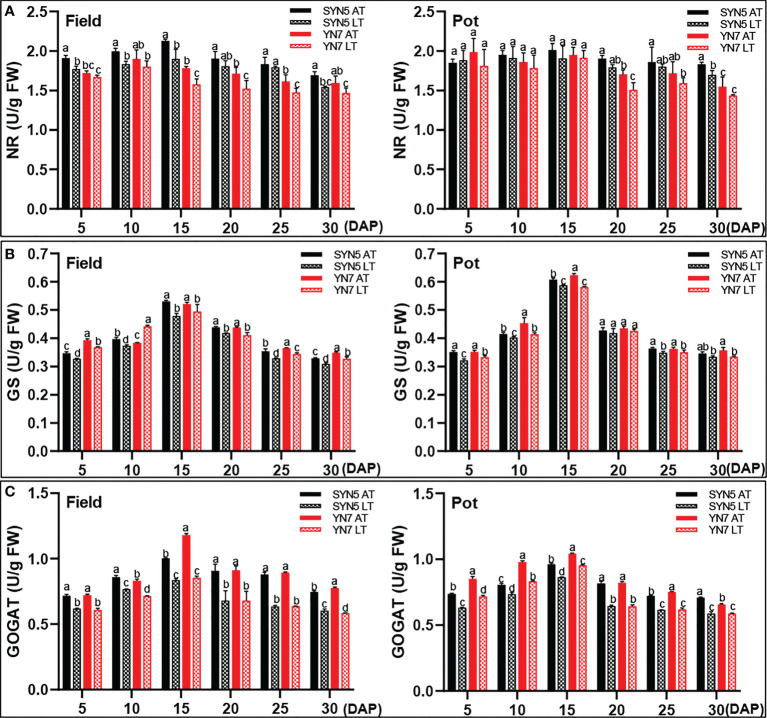
Effect of post-silking LT on the enzymatic activities of protein synthase in grains during grain filling stage. **(A)**, GS (glutamine synthetase); **(B)**, GOGAT (glutamate synthase); **(C)**, NR (nitrate reductase). Error bars denote standard errors from three replicates, and different letters above the bars in same sampling date are significantly different at *P* < 0.05.

Compared with the AT treatment, the LT treatment significantly decreased the activity of NR at 5, 10, 15, and 30 DAP in SYN5 and 10 DAP in YN7 in the field trial (*P* < 0.05; **Figure 5A**). However, the LT treatment exerted no effect on NR activity in both varieties in the pot trial. LT treatment also significantly decreased the activity of GS in both varieties in the field trial (*P* < 0.05) (**Figure 5B**). In the pot trial, the LT treatment decreased GS activity during kernel development, except for 10, 20, and 30 DAP of SYN5 and 20 DAP of YN7. Meanwhile, LT treatment significantly reduced the activity of GOGAT in both waxy maize varieties and both experimental environments (*P* < 0.05). The reduction of GOGAT activity was greater in YN7 than in SYN5 (**Figure 5C**).

### Effect of low temperature on the contents of endogenous hormones in grains

**Figure 6:**
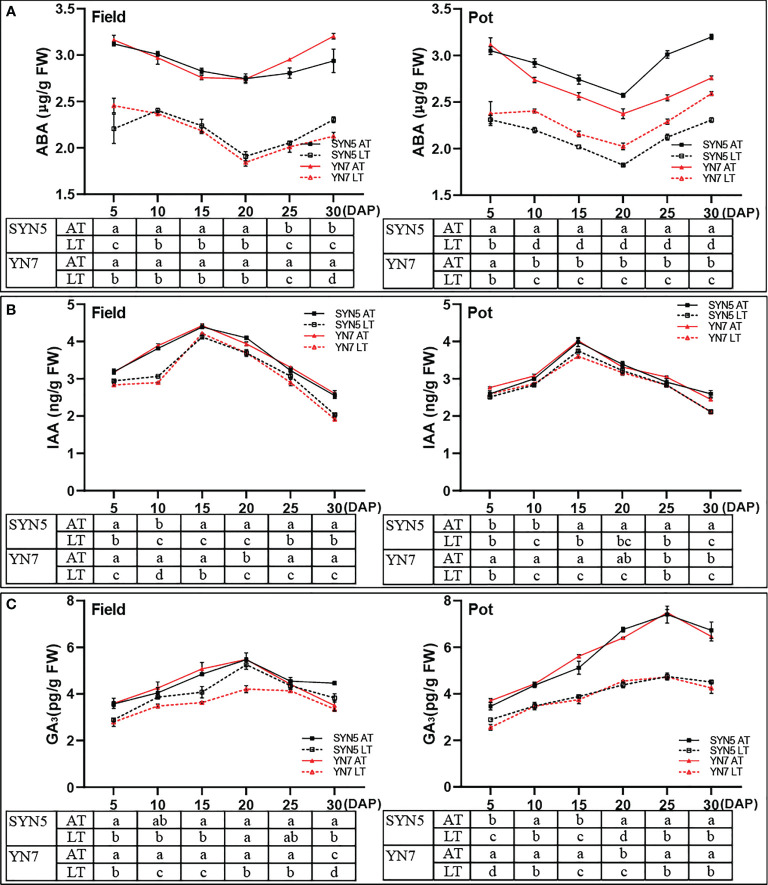
Effect of post-silking LT on the contents of endogenous hormones in grains during grain filling stage. **(A)**, ABA (abscisic acid); **(B)**, IAA (indole-3-acetic acid); **(C)**, GA_3_ (gibberellin 3). Error bars denote standard errors from three replicates, and different letters at the same sampling date indicate significant difference at *P* < 0.05.

The ABA content of all treatments initially decreased and then gradually increased with grain filling in both varieties and experimental environments and was the lowest at 20 DAP (**Figure 6A**). By contrast, the IAA and GA_3_ contents in the grains initially increased and then decreased with the kernel development under AT and LT treatments in both varieties and experimental environments (**Figures 6B, C**). Compared with the AT treatment, the LT treatment significantly decreased the ABA, IAA, and GA_3_ contents in both varieties and experimental environments (*P* < 0.05). On average, the decline rates of ABA, IAA, and GA_3_ in grains were greater during the kernel development of YN7 than during the kernel development of SYN5 under the LT treatment.

### Effects of low temperature on flour pasting properties

The LT treatment significantly decreased the PV, TV, and FV of grains compared with the AT treatment in both varieties and experimental environments (*P* < 0.05). The BD of SYN5 was increased under LT treatment in both experimental environments compared with the AT treatment, whereas the opposite was observed in YN7 (*P* < 0.05). Compared with the AT treatment, the SB of both varieties increased under LT treatment in the field trial but decreased in the pot trial. The LT treatment significantly reduced the *P*_temp_ of SYN5 in both experimental environments (*P* < 0.05). Meanwhile, the PV, TV, BD, FV, and SB of grains were lower in the field trial than in the pot trial in both varieties, and no effect was observed on *P*_temp_ (*P* < 0.05).

### Effects of low temperature on flour thermal properties

Compared with the AT treatment, the LT treatment significantly reduced the Δ*H*_gel_ of grains in both varieties and trials (*P* < 0.05). The LT treatment significantly decreased *T*_o_ in the field trial of SYN5 and the pot trial of both varieties (*P* < 0.05). In addition, the LT treatment significantly increased *T*_p_ in the field trial of SYN5 and decreased it in the pot trial of both varieties (*P* < 0.05). The LT treatment significantly decreased the *T*_c_ of YN7 in both trials and that of SYN5 in the pot trial. The Δ*H*_ret_ under the LT treatment increased in SYN5 in the field trial and decreased in both varieties in the pot trial compared to that under the AT treatment. The %*R* under the LT treatment was higher in both varieties in the field trial than in the AT treatment. In addition, the Δ*H*_gel_, *T*_p_, *T*_o_, and *T*_c_ in the pot trial were lower than those in the field trial. By contrast, the %*R* in the pot trial was higher than that in the field trial, except for SYN5 under the LT treatment.

## Discussion

LT during the grain filling stages causes great yield loss of cereal crops (Gao Z. et al., 2018; Huang et al., 2021). Ji et al. (2017) investigated the effects of LT stress at the booting stage on wheat yield and found that the grain yield and spike number per plant and the grain number per spike significantly reduce under LT stress. In the present study, the kernel fresh and dry weights of both waxy maize varieties were reduced under the post-silking LT treatment during kernel development (**Figure 2**). LT during the grain filling stage mainly reduces the filling rate and delays the filling process, resulting in insufficient grain dry matter accumulation and reduced grain weight (Ali et al., 2021). A previous study has found that LT can damage the green leaves of plants, thereby reducing the source and content of assimilates, and weakening the transportation and distribution of assimilates (Ying et al., 2000). Furthermore, LT may also reduce the assimilate capacity of kernel sink by restricting grain development (Zhao et al., 2022). The change of source-sink relationships under LT stress leads to the decrease of grain filling rate and grain weight (Huang et al., 2019; Huang et al., 2021). In this study, Pearson’s correlation analysis showed that the accumulation of soluble sugar, starch, and protein were positively correlated with the changes in of fresh and dry weight of grain (**Figure S1**), which was consistent with previous studies (Zhang et al., 2021; Zhao et al., 2022). Changes of grain yield under LT stress were also affected by cellular carbohydrates, lipids, cell viability, and gene expression (Laudencia-Chingcuanco et al., 2011; Skinner et al., 2014). The decrease in the kernel weight of YN7 under the LT treatment was greater than that of SYN5, indicating that YN7 was more sensitive than SYN5.

Starch and protein biosynthesis and accumulation are significantly influenced by genetic and environmental factors (Zhang et al., 2021). This study proved that post-silking LT treatment significantly decreased the accumulation of soluble sugar and starch during grain filling (**Figure 3**). The decrease of sugar and starch contents in grains under LT treatment may be related to the decrease of photosynthetic rate and assimilation accumulation (Zeng et al., 2011; Zhang et al., 2015). In starch and protein syntheses, various enzymes participate in the metabolism of carbohydrates and nitrogen during kernel development in cereal crops (Marschner, 2011; Dong and Beckles, 2019). The activities of SPS, AGPase, SuSy, SSS, and SBE decreased significantly during grain filling in late sown date (Mahla et al., 2017), and similar results was found in this research (**Figure 4**). The reduction of these enzymatic activities leads to a decrease in the conversion of starch synthesis substrates and starch accumulation efficiency (Zhang et al., 2018). Additionally, LT also reduced the starch biosynthesis rate and starch content by down-regulating the expression of starch synthesis-related genes, thus inhibiting the grain filling rate (Xu et al., 2021). In this study, Pearson’s correlation analysis further revealed that the enzymatic activities related to starch synthesis were positively correlated with starch content, starch accumulation, and dry matter accumulation in grain (**Figure S1**). LT stress also reduces total aboveground N and increases the rate of grain N accumulation (Huang et al., 2021). In the present study, LT stress increased the protein content at the maturity stage but decreased the protein accumulation during grain filling (**Figure 3**). The relative increase in protein content is due to a large reduction in grain starch (Seebauer et al., 2010; Akter and Islam, 2017). Another reason is that the increase of protein content in the grain may be a compensatory mechanism to offset the decrease of starch content (Wang et al., 2018). LT stress reduced the activities of GOGAT and GS in both trials and NR in the field trial during grain filling (**Figure 5**), resulting in a decrease in protein accumulation (**Figure S1**). GS/GOGAT cycles are the first step of ammonium assimilation in higher plants, and the reduction of their activities directly affects N uptake, translocation, remobilization, and amino acids conversion (Miflin and Habash, 2002). Thus, LT stress may decrease the production and supply of protein synthesis substrates in the grains by reducing N utilization efficiency (Andersson and Holm, 2011). In the present study, the effect of LT on the enzymatic activities related to starch and protein synthesis of YN7 was greater than that of SYN5, which indicated that SYN5 has a strong LT tolerance.

Phytohormones, as signaling factors under LT stress, play important roles in regulating balance (Zhang et al., 2019). The changes and interactions of various phytohormones in the grains affect grain development and grain filling process (Gao J. et al., 2018). Zhang et al. (2019) found that LT stress inhibited ears development and grain yield formation of wheat by increasing ABA contents and reducing IAA and GA contents in spikelets. Previous studies suggested that ABA in grains is a key factor regulating the expression of starch synthesis genes and grain filling under abiotic stresses (Yang et al., 2004; Tsukaguchi et al., 2018). In the present study, post-silking LT treatment decreased the ABA content during grain filling in both varieties and environments (**Figure 6**). The decrease of ABA content can limit the division of endosperm cells, reduce the storage capacity and grain filling rate (Huang et al., 2016). IAA promotes grain filling and grain size initiation by regulating endosperm cell division and expansion (Yu et al., 2020). A decrease of IAA level in endosperm induced by temperature stress leads to abnormal grain filling, lower grain mass in inferior spikelets, and reduced grain weight of rice (Cao et al., 2016). Similarly, post-silking LT treatment reduced the IAA content during kernel development (**Figure 6**), which may affect the transport of assimilates to the developing kernels and starch accumulation (Lur and Setter, 1993; Abu-Zaitoon et al., 2012). GA_3_ is one of the important hormone that regulate the grain filling rate and duration (Zhang et al., 2019). It is reported that LT stress reduces GA_3_ content in pollen and spikelet, which results in a decrease in rice and wheat yield (Sakata et al., 2014; Zhang et al., 2019). In the present study, the content of GA_3_ during kernel development significantly decreased under the LT treatment in both varieties and environments (**Figure 6**). The decrease of GA_3_ content under LT treatment may affect grain yield by inhibiting embryogenesis and embryo enlargement (Zhang et al., 2016). These results suggest that the growth and development of grain depends not only on the regulation of one hormone type but also on the balance among various hormones. Correlation analysis further indicated that the reciprocal regulation between endogenous hormones and enzymes jointly regulates the synthesis and accumulation of starch and protein in grains (**Figure S1**). Furthermore, the decrease in ABA, IAA, and GA_3_ contents caused by the LT treatment in YN7 was greater than that of SYN5, suggesting that growth inhibition was more severe in YN7 than in SYN5.

Gelatinization and retrogradation are important indicators to evaluate the physicochemical and functional properties of flour (Ketthaisong et al., 2013; Bhat and Riar, 2019). Previous studies indicated that the occurrence of temperature stress during the grain filling of wheat, rice, and waxy maize would greatly affect the grain plumpness and grain functional properties (Lu and Lu, 2013; Hu et al., 2020; Zhang et al., 2021). Hu et al. (2020) suggested that LT stress during grain filling increases amylose content in grains of both rice varieties, reduces thermal properties (*T*_o_, *T*_p_, and *T*_c_) and pasting properties (PV, TV, FV, and BD), and decreases eating quality of rice. In the present study, similar declines in PV, TV, FV, and *P*_temp_ in flour were observed following two environmental LT stresses in both varieties (**Table 1**). Our previous study found that flour pasting properties are positively correlated with starch content in grains and negatively correlated with protein content and starch grain size (Wen et al., 2019), and this study also found similar results (**Figure S2**). The increase of protein content under LT stress will limit the swelling of starch in flour under heating, resulting in a decrease in flour pasting properties (Noisuwan et al., 2008). Additionally, the deficiency of starch synthesis-related enzymatic activities and the reduction of starch content under LT stress may also inhibit flour swelling (Bhat and Riar, 2019; He and Wei, 2020; Yang H. et al., 2021). LT stress decreased the Δ*H*_gel_, *T*_o_, *T*_p_, *T*_c_, and Δ*H*_ret_ in both varieties in pot trial and Δ*H*_gel_ in both varieties in field trial (**Table 2**). Correlation results indicated that Δ*H*_gel_, *T*_p_, *T*_o_, and *T*_c_ positively correlated with starch content and negatively correlated with protein content at maturity in both varieties (**Figure S2**). The low gelatinization temperatures may be due to LT stress restricting endosperm cell division and the number of amyloplasts, resulting in a decrease in starch content and an increase in starch granule size, which results in poorer structure stability of flour (Li et al., 2010; Wani et al., 2012; Jing et al., 2014). The increase in starch granule volume interferes with the formation of gel network during flour retrogradation (Martinez et al., 2018; Li et al., 2020). LT treatment increases the %*R* of flour because the high protein content easily forms matrix during sample cooling and more energy is required for reheating (Noisuwan et al., 2008). Thus, the gelatinization and retrogradation properties of flour are closely related to starch and protein metabolism. Compared with YN7, SYN5 has a higher viscosity, which indicates that it has an advantage in products that require high viscosity. The %*R* of SYN5 was higher than that of YN7 under LT treatment, indicating that the starch of YN7 is more dominant than SYN5 in retrograde tendency. The differences in the functional properties of SNY5 and YN7 grains may be related to their grain component content (Lu et al., 2014b; Wen et al., 2019). Therefore, the present study suggests that post-silking LT stress not only affects physiological and biochemical characteristics but also ultimately affects grain quality.

**Table 1:** Effects of post-silking low temperature on flour pasting properties of waxy maize.

Trial	Variety	Treatment	PV (mPa.s)	TV (mPa.s)	BD (mPa.s)	FV (mPa.s)	SB (mPa.s)	*P*_temp_(°C)
Field	SYN5	AT	741.7 ± 16.5d	727.7 ± 20.1c	16.3 ± 0.6fg	975.3 ± 8.1c	247.7 ± 13.7b	77.3 ± 0.5c
		LT	524.0 ± 17.4e	467.7 ± 29.3g	48.0 ± 5.6c	620.3 ± 38.6g	152.7 ± 9.3d	75.4 ± 0.5d
	YN7	AT	718.7 ± 14.7d	619.3 ± 22.5e	35.0 ± 2.0d	923.7 ± 11.5d	371.0 ± 10.3a	76.2 ± 1.7cd
		LT	521.7 ± 13.0e	509.7 ± 6.1f	15.3 ± 5.0g	682.3 ± 2.5f	172.7 ± 4.6cd	75.7 ± 0.9d
Pot	SYN5	AT	864.7 ± 21.9b	845.0 ± 17.1a	23.0 ± 1.0ef	1085.0 ± 7.0a	230.0 ± 4.0c	81.6 ± 0.1a
		LT	814.7 ± 16.8c	788.3 ± 25.8b	29.7 ± 3.5de	1046.7 ± 28.1b	258.3 ± 5.0b	78.0 ± 0.9bc
	YN7	AT	895.7 ± 6.7a	785.3 ± 12.5b	110.3 ± 7.2a	952.7 ± 15.1cd	167.3 ± 5.7cd	78.6 ± 0.5b
		LT	730.3 ± 26.1d	668.7 ± 25.0d	61.7 ± 3.1b	841.0 ± 25.0e	172.3 ± 3.1cd	77.8 ± 0.5b

PV, peak viscosity; TV, trough viscosity; BD, breakdown viscosity; FV, final viscosity; SB, setback viscosity; *P*_temp_, pasting temperature; mPa.s, centipoise. Mean values in the same column followed by different letters are significantly different (*P* < 0.05).

**Table 2:** Effects of post-silking low temperature on flour thermal properties of waxy maize.

Trial	Variety	Treatment	Δ*H*_gel_ (J/g)	*T*_o_ (°C)	*T*_p_ (°C)	*T*_c_ (°C)	Δ*H*_ret_ (J/g)	%*R* (%)
Field	SYN5	AT	7.4 ± 0.2b	68.9 ± 0.6b	75.9 ± 0.7c	82.4 ± 0.7b	2.8 ± 0.0c	38.2 ± 0.8cd
		LT	6.8 ± 0.0cd	66.0 ± 0.7c	77.9 ± 0.5b	83.0 ± 0.1b	3.3 ± 0.2ab	47.5 ± 2.8a
	YN7	AT	7.3 ± 0.2bc	70.7 ± 0.3a	79.8 ± 1.3a	85.8 ± 0.9a	2.5 ± 0.1c	35.2 ± 1.3d
		LT	6.8 ± 0.0de	72.1 ± 0.7a	78.4 ± 0.2ab	83.7 ± 0.4b	2.7 ± 0.0c	39.9 ± 0.8c
Pot	SYN5	AT	7.1 ± 0.2bcd	67.7 ± 1.7b	73.8 ± 1.0d	82.0 ± 1.4b	3.3 ± 0.3ab	45.6 ± 4.2ab
		LT	6.4 ± 0.2ef	65.1 ± 0.8cd	71.1 ± 1.5e	79.4 ± 1.3c	2.7 ± 0.2c	41.4 ± 3.1bc
	YN7	AT	7.9 ± 0.4a	64.3 ± 0.8de	71.3 ± 0.7e	80.3 ± 1.3c	3.4 ± 0.5a	42.7 ± 4.7bc
		LT	6.3 ± 0.8f	63.0 ± 0.6e	69.4 ± 0.9f	77.0 ± 0.6d	2.9 ± 0.2bc	45.7 ± 0.7ab

Δ*H*_gel_, gelatinization enthalpy; *T*_o_, onset temperature; *T*_p_, peak gelatinization temperature; *T*_c_, conclusion temperature; Δ*H*_ret_, retrogradation enthalpy; %*R*, retrogradation percentage. Mean values in the same column followed by different letters are significantly different (*P* < 0.05).

Sowing time experiments is an important means to explore the effects of temperature changes in natural environments (Isobe et al., 2022). Meanwhile, the pot trial can strictly control the growth environment of plants, which is beneficial to accurately determine the influence of experimental factors (Gheysari et al., 2021). Previous study have shown that the results from pot trial can not only explain and verify the findings from field trial, but also help to explore the physiological and biochemical mechanisms (Li et al., 2019). In the present study, the effects of LT during grain filling stage on the physiological, biochemical and functional properties of the two waxy maize varieties showed the same trend in both pot and field trials, and the results of these two trials were mutually verified. Additionally, compared with the pot trail, the field trial had a greater effect on the carbon and nitrogen metabolism-related enzymatic activities, hormones contents, starch content and yield. The reasons for the difference between the two experiments are (1) the temperature at the second sowing date in the field is lower than that in the greenhouse (**Figure 1**), and (2) the environment of the field trial is more complicated than that of the pot trial. Overall, multi-environmental experiments can more accurately explore the effects of LT stress on grain development.

## Conclusion

Post-silking LT stress has significant negative effects on grain filling process and dry matter accumulation in waxy maize grains during grain filling. LT stress reduced the accumulation of starch and protein and yield by reducing the activities of key starch and protein synthesis enzymes and the contents of various endogenous hormones during grain filling. Changes in starch and protein contents under LT treatment in pot and field trials decreased the PV, TV, FV, *P*_temp_, and Δ*H*_gel_, and increased the %*R*, which ultimately changed the grain quality. Meanwhile, the results of the two environments were slightly different, which may be that the environment of the field is more complex than that of the controlled climatic chambers. Furthermore, physiological and biochemical characteristics of grain indicated that SYN5 had stronger cold tolerance than YN7. Our findings suggest that grain filling process and quality formation in waxy maize grains under LT stress were affected by complex regulatory mechanisms. Therefore, this study could generally reflect the effects of post-silking LT on the grain development and quality formation of waxy maize.

## Data availability statement

The original contributions presented in the study are included in the article/**Supplementary Material**. Further inquiries can be directed to the corresponding author.

## Author contributions

DL conceived and designed the experiments and revised the manuscript. QW managed the experiments and took the measurements. JG and LQ analyzed and interpreted the data. JG wrote the first draft of the manuscript. All authors contributed to the article and approved the submitted version.

## Funding

This study was supported by the National Natural Science Foundation of China (31771709, 32071958), Jiangsu Agricultural Industry Technology System (JATS[2022]497), Jiangsu Agriculture Science and Technology Innovation Fund (CX[20]3147), Key Research & Development Program of Jiangsu Province (BE2021317), Priority Academic Program Development of Jiangsu Higher Education Institutions, and High−end Talent Support Program of Yangzhou University.

## Conflict of interest

The authors declare that the research was conducted in the absence of any commercial or financial relationships that could be construed as a potential conflict of interest.

## Publisher’s note

All claims expressed in this article are solely those of the authors and do not necessarily represent those of their affiliated organizations, or those of the publisher, the editors and the reviewers. Any product that may be evaluated in this article, or claim that may be made by its manufacturer, is not guaranteed or endorsed by the publisher.

## Supplementary material

Supplementary Figure 1 Heatmap of correlation coefficients between kernel weight, grain component content and accumulation, starch and protein synthesis-related enzymatic activity, and endogenous hormone content under LT treatment. **(A)**, SYN5; **(B)**, YN7. Circles of different sizes reflect the correlation coefficient. Red represents negative correlation and blue represents positive correlation. KFW, kernel fresh weigh; KDW, kernel dry weight; SUC, soluble sugar content; SUA, soluble sugar accumulation; STC, starch content; STA, starch accumulation; PRC, protein content; PRA, protein accumulation; SuSy, sucrose synthase; SPS, sucrose phosphate synthase; AGPase, ADP-glucose pyrophosphorylase; SSS, soluble starch synthase; SBE, starch branching enzyme; SDBE, starch-debranching enzyme; GS, glutamine synthetase; GOGAT, glutamate synthase; NR, nitrate reductase; ABA, abscisic acid; IAA, indole-3-acetic acid; GA_3_, gibberellin 3. The correlation analysis was performed in triplicate (n = 3).

Supplementary Figure 2 Heatmap of correlation coefficients between pasting properties, thermal properties, starch and protein contents at maturity under LT treatment. **(A)**, SYN5; **(B)**, YN7. Circles of different sizes reflect the correlation coefficient. Red represents negative correlation and blue represents positive correlation. MSC, starch content at maturity; MPC, protein content at maturity; PV, peak viscosity; TV, trough viscosity; BD, breakdown viscosity; FV, final viscosity; SB, setback viscosity; *P*_temp_, pasting temperature; Δ*H*_gel_, gelatinization enthalpy; *T*_o_, onset temperature; *T*_p_, peak gelatinization temperature; *T*_c_, conclusion temperature; Δ*H*_ret_, retrogradation enthalpy; %*R*, retrogradation percentage. The correlation analysis was performed in triplicate (n = 3).
