# Supplementary figures and images for: Effects of post-silking low temperature on the starch and protein metabolism, endogenous hormone contents, and quality of grains in waxy maize
Source: Front Plant Sci. 2022 Nov 4;13:988172. doi: 10.3389/fpls.2022.988172 (PMC9673756; doi:10.3389/fpls.2022.988172)

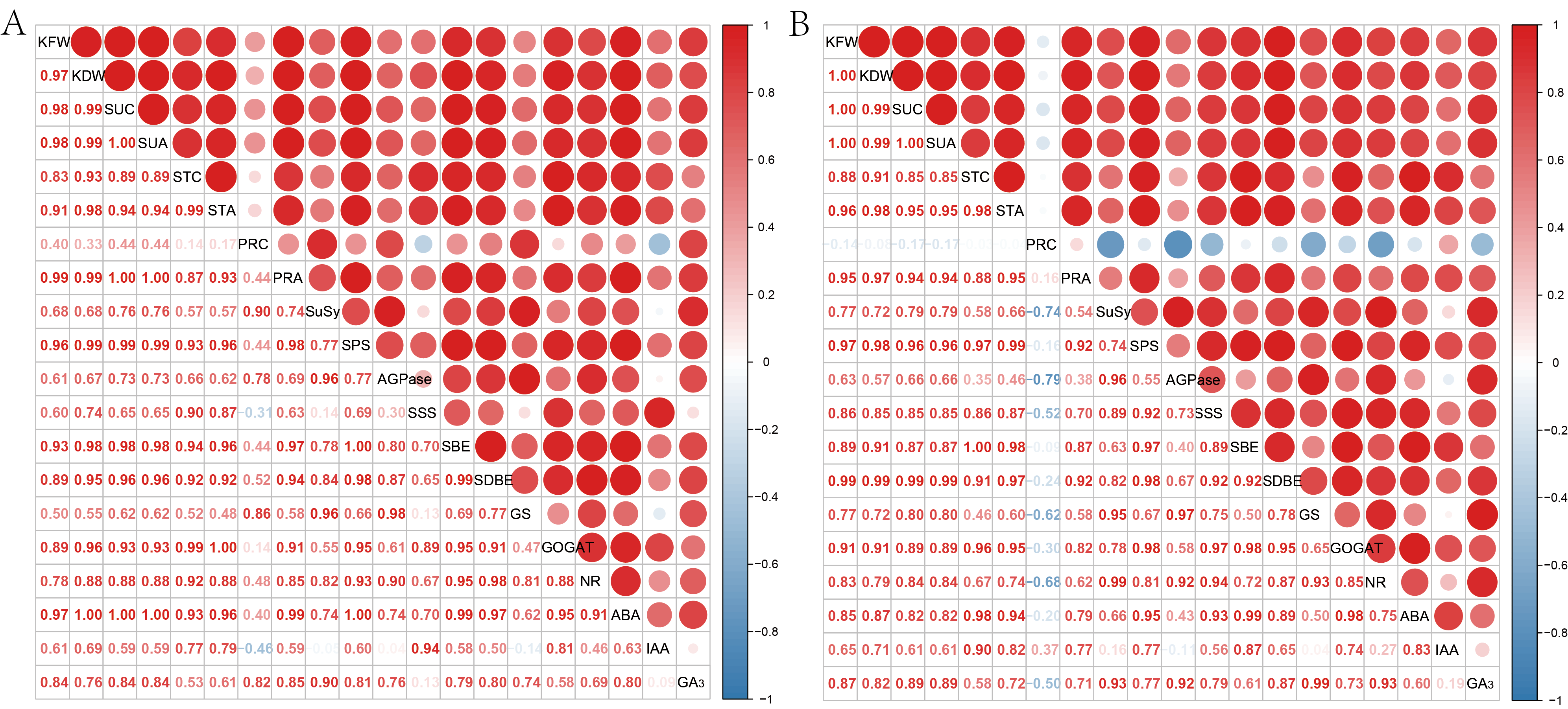

Supplement: Supplementary Figure 1 — Heatmap of correlation coefficients between kernel weight, grain component content and accumulation, starch and protein synthesis-related enzymatic activity, and endogenous hormone content under LT treatment. (A), SYN5; (B), YN7. Circles of different sizes reflect the correlation coefficient. Red represents negative correlation and blue represents positive correlation. KFW, kernel fresh weigh; KDW, kernel dry weight; SUC, soluble sugar content; SUA, soluble sugar accumulation; STC, starch content; STA, starch accumulation; PRC, protein content; PRA, protein accumulation; SuSy, sucrose synthase; SPS, sucrose phosphate synthase; AGPase, ADP-glucose pyrophosphorylase; SSS, soluble starch synthase; SBE, starch branching enzyme; SDBE, starch-debranching enzyme; GS, glutamine synthetase; GOGAT, glutamate synthase; NR, nitrate reductase; ABA, abscisic acid; IAA, indole-3-acetic acid; GA3, gibberellin 3. The correlation analysis was performed in triplicate (n = 3). [file Image_1.tif]

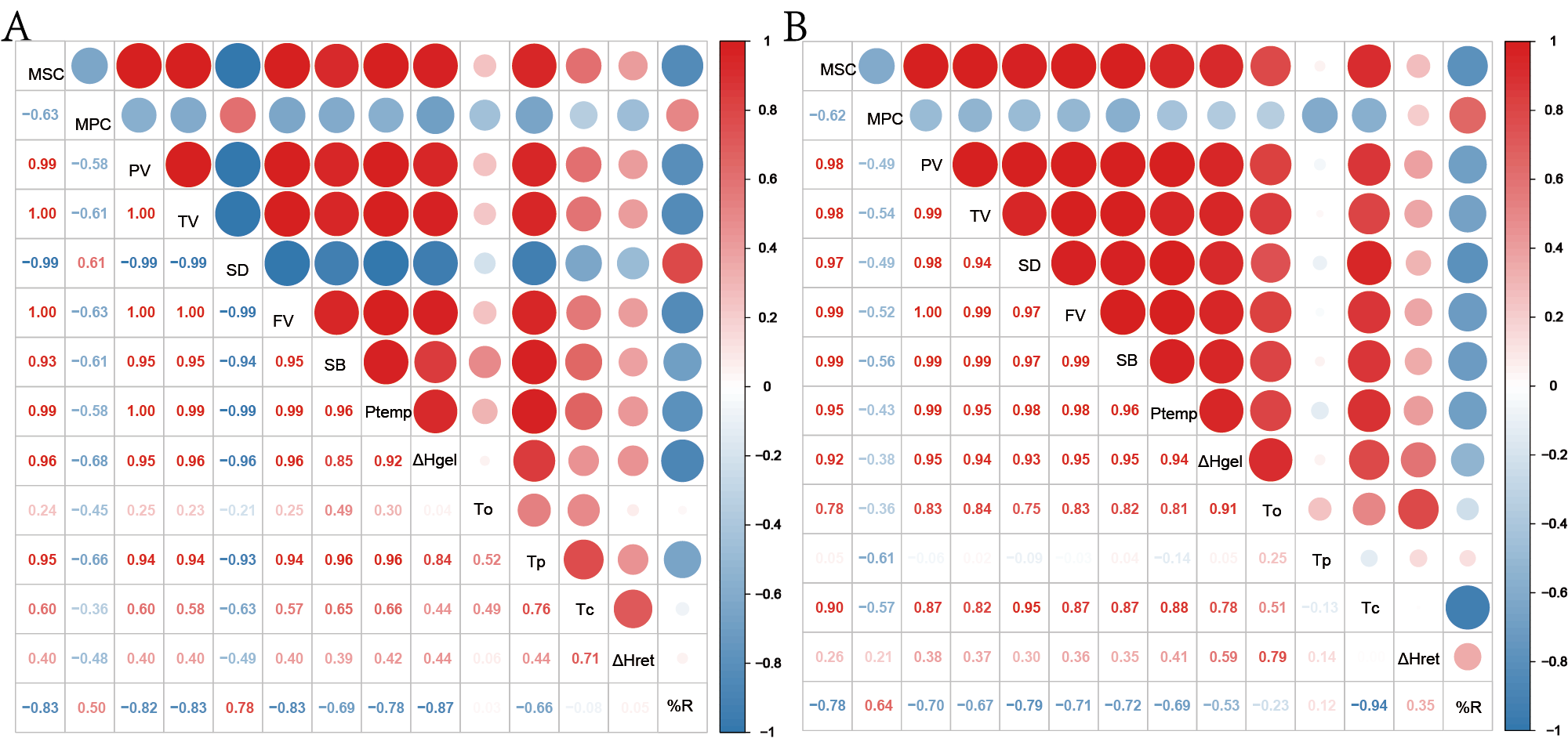

Supplement: Supplementary Figure 2 — Heatmap of correlation coefficients between pasting properties, thermal properties, starch and protein contents at maturity under LT treatment. (A), SYN5; (B), YN7. Circles of different sizes reflect the correlation coefficient. Red represents negative correlation and blue represents positive correlation. MSC, starch content at maturity; MPC, protein content at maturity; PV, peak viscosity; TV, trough viscosity; BD, breakdown viscosity; FV, final viscosity; SB, setback viscosity; P temp, pasting temperature; ΔH gel, gelatinization enthalpy; T o, onset temperature; T p, peak gelatinization temperature; T c, conclusion temperature; ΔH ret, retrogradation enthalpy; %R, retrogradation percentage. The correlation analysis was performed in triplicate (n = 3). [file Image_2.tif]
